# Mitigating security flaws in Baptista’s chaotic cryptosystem through superior and alternated logistic map approaches

**DOI:** 10.1038/s41598-025-22269-8

**Published:** 2025-11-03

**Authors:** Deepak Kumar Verma, Mamta Rani, Roli Dikshit, Himanshu Gupta, Anuj Kumar

**Affiliations:** 1 Department of Computer Engineering, Marwadi University, Rajkot, 360006 Gujarat India; 2 Department of Computer Science, Central University of Rajasthan, Kishangarh, Ajmer, 305817 Rajasthan India; 3 Department of Instrumentation and Control Engineering, Manipal Institute of Technology, Manipal Academy of Higher Education, Manipal, 576104 Karnataka India; 4 Department of Design, Data Science and Cyber Security, Greater Noida Institute of Technology, Greater Noida, 201310 India

**Keywords:** Baptista chaotic cryptosystem, Superior orbit, Logistic map, Key recovery attacks, Entropy attack, And one-time pad attack, Computational science, Computer science

## Abstract

This research delves into the concerns raised by numerous researchers regarding the security of Baptista’s chaotic cryptosystem. The paper scrutinizes two fundamental issues in the Baptista algorithm: the imperfect distribution of chaotic data and the constraints of a single attractor. To address these issues, we propose a novel approach that leverages Parrondo’s paradox to switch between two chaotic attractors and integrates a more robust logistic map into the Baptista algorithm by introducing additional parameters to the secret key. The two innovative methods, Superior Baptista and alternated Superior Baptista cryptosystems, are proposed to yield improved chaotic data or two new chaotic attractors. Therefore, the security features of the cryptosystem are enhanced by increasing the number of system iterations used for encryption. Experimental evaluations show that the Superior Baptista Algorithm required iteration counts up to 2,823, while the Alternated variant reached 86,556 iterations for specific characters, significantly improving randomness and diffusion. Furthermore, the entropy values of these algorithms are calculated 67.35% and 42.86% higher than those of the Baptista algorithm, respectively. These findings demonstrate the superiority of the proposed cryptosystems over the base algorithm.

**Supplementary Information:**

The online version contains supplementary material available at 10.1038/s41598-025-22269-8.

## Introduction

Data security is becoming a significant concern to industries such as healthcare, finance, and military. Conventional cryptographic methods are not efficient in mitigating data security concerns due to the dynamic nature of threats^1^. Chaotic systems gained popularity for securing confidential data following the expansion of internet usage for data transmission and storage. Their inherent dynamical traits, sensitivity to initial conditions, exponential divergence, ergodicity, and mixing, make them well-suited for cryptographic purposes. Typically, a chaotic generator relies on uncomplicated nonlinear recursive equations. While such equations may appear tame, they defy convergence to any fixed point or invariant set, thus ensuring unpredictability even after countless iterations^2^.

Chaotic cryptography has since evolved into an active area of research. Scholars have adopted chaotic signals over the past forty years to protect analog and digital transmissions. The hallmark of such schemes is their dependency on initial states and system parameters, which produce sequences that mimic truly random noise. An observer can regenerate the sequence precisely if the secret parameters are identical. Among the many constructions, Baptista-type algorithms predominantly rely on the logistic map to obfuscate textual^3–5^. However, Baptista’s cryptosystems struggle due to several vulnerabilities (for example, non-uniform ciphertext distribution and limited key space), which limit their practical utility and motivate improvements in chaotic encryption frameworks to meet modern security demands.

### Motivation and contributions

The logistic maps are efficiently used to encrypt Baptista’s chaos-based chaotic cryptosystems. They suffer from notable limitations, such as non-uniform ciphertext distribution, limited key space, and susceptibility to known attack models^4,6^. These limitations prompted researchers to explore enhancements such as dynamic lookup tables and multi-dimensional chaotic systems^7–9^. Yet, most of these models continued to rely on a single chaotic attractor, leaving them vulnerable to key recovery and entropy-based attacks due to predictable orbit patterns or non-uniform distribution^10^. Furthermore, improvements often neglect the trade-off between encryption strength and computational feasibility. These gaps highlight the need for a system that balances enhanced security, improved randomness, and practical implementation.

To address these gaps, the proposed work builds upon Baptista’s foundational model. Still, it introduces two major innovations: (i) the integration of Superior logistic maps (via a Superior orbit mechanism), and (ii) the deterministic alternation of chaotic parameters inspired by Parrondo’s paradox. Superior orbits, defined by a tunable parameter *β*, yield a more uniformly distributed chaotic sequence and expand the secret key space, thereby directly mitigating the non-uniformity problem highlighted in literature^4,6^. The alternate Superior logistic map further enhances security by employing two logistic maps (*r*₁ and *r*₂) in an alternating sequence, adding unpredictability and increasing resistance to ciphertext-only and chosen-plaintext attacks^11^.

These mechanisms not only improve encryption complexity and robustness but also demonstrably increase iteration depth, effectively enhancing diffusion properties. Additionally, our approach retains computational feasibility while enhancing security performance against classical attack models, a feature that has been underexplored in earlier enhancements^3,12,13^. Summarizing, the key contributions of the present work can be highlighted as:

1. Proposes the use of Superior logistic maps with a tunable parameter β to improve the uniformity of the chaotic sequence and expand the key space, thus enhancing resistance against entropy- and distribution-based attacks.
2. Employs an alternating sequence of logistic maps (*r*_1_, *r*_2_) to increase randomness and unpredictability, inspired by Parrondo’s paradox. This method disrupts pattern formation and enhances security against ciphertext-only and chosen-plaintext attacks.
3. Strengthens the diffusion process by increasing the unpredictability and spread of ciphertext changes through deeper and more diverse orbit traversal through Superior orbits.
4. Provides a better balance between encryption strength and computational feasibility, making it suitable for deployment in real-time applications.
5. Conducts an extensive performance evaluation and comparison of the proposed scheme against the existing models in terms of security metrics, randomness, and computational complexity.

### Organization

The remainder of this paper is organized as follows: In section “Related work”, we survey the literature on chaos-based cryptography, summarizing earlier techniques and their identified vulnerabilities. Section “Preliminaries” reviews the foundational material, presenting the logistic mapping, properties of chaotic dynamical systems, and the innovative Superior Orbit mechanism. Section “Proposed Baptista algorithm in superior orbit” formulates the Baptista Algorithm, endowed with a Superior Orbit, and explicates the underlying equations, key management, and the workflow from plaintext to ciphertext. Empirical findings, including entropy metrics, the adequate key space, and assessments of cryptanalytic resistance are reported in section “Results and discussions”. In section “The vulnerability analysis”, vulnerability analysis of Baptista and the proposed enhancements are discussed. Section “Limitations” presents the limitations of the proposed work. Finally, section “Conclusion” concludes the work with possible outlines for future investigation.

## Related work

Motivated by the limitations described in section “Introduction”, we review previous work that attempted to enhance Baptista’s cryptosystem in this section. Baptista’s Chaotic System, utilizing Superior logistic maps, represents an exciting step forward in the field of cryptography, particularly in enhancing communication security. The chaotic nature of logistic maps is an ideal toolkit for generating pseudo-random sequences necessary for encryption. Recently, the benefits of using chaotic systems with cryptographic impact have been highlighted^14,15^. These features are significant for secure encryption due to their unpredictability and oversensitivity to changes in initial conditions. The application of chaotic maps, especially Superior logistic maps, has already significantly impacted the construction of cryptographic systems by enhancing the security and complexity of encryption algorithms. For example, it has been demonstrated that chaotic maps can improve the diffusion and confusion of the encryption schema, making it more robust against different types of cryptanalytic attacks^16^. More advanced chaotic systems enable the creation of sophisticated key streams that are more difficult to penetrate, thereby protecting both the data and its transmission. In addition, some selected empirical research has confirmed the use of Superior logistic maps in Baptista’s system. These studies demonstrate that the random behavior of logistic maps enhances the randomness of the generated keys, which is used to counter possible attacks, such as chosen plaintext or ciphertext attacks. These maps are also applied in telemedicine-related fields, such as medical image encryption and other sensitive communications. Moreover, the construction of hybrid encryption schemes that incorporate chaotic maps with other encryption techniques is quite promising and is currently under active research. For example, in^16^, chaotic maps were incorporated into DNA encoding to enhance security in digital healthcare applications. In^17^, the use of chaotic systems for novel and efficient encryption techniques was further explored, highlighting their efficacy in addressing new and complex threats to modern communication systems over time.

Baptista’s Chaotic Cryptosystem, employing Enhanced Logistic Maps, is a notable contribution to the field of cryptography, particularly in the areas of image encryption and data protection. The logistic map’s chaotic behavior is a robust method for generating pseudo-random sequences, which is critical for sophisticated cryptographic systems. Many researchers have recently focused on using chaotic maps in cryptography for their output’s discernible intricacies and unpredictability, making system undermining nearly impossible. For example,^18^ demonstrated that combining a perturbed one-dimensional logistic map with a logistic map significantly enhances the randomness and security of standard precision device algorithms, thereby mitigating some of the security weaknesses inherent in low-precision devices. In addition, such chaos-based encryption improved data transmission efficiency alongside the system’s security. The modifications to the Chacha20 algorithm using logistic maps have been demonstrated in^19^, which reveals that these chaotic systems can accelerate and enhance encryption security without compromising the balance between precision and execution time.

Furthermore, the effectiveness of chaotic maps for cryptosystems was evaluated by developing a fast color image encryption algorithm that relies on S-boxes and hyperchaotic maps^20^. It is worth mentioning that the chaos traits in logistic maps often aid in the diffusion processes, which form an intricate part of the confusion processes and are essential in cryptographic algorithms. Many researchers have pointed out that medical image encryption has benefited from chaos due to the strength it lends to encryption techniques through the use of chaos maps. These have immense relevance to fields such as medicine and finance, where data protection is a focal point. The Superior logistic maps at Baptista’s Chaotic Cryptosystem are used to build a high-level security system for encryption. The integration of chaotic systems into traditional systems enhances security, which addresses the complexity issues associated with non-standard encryption techniques. In chaotic cryptography, the plaintext is converted into a ciphertext-like code^21^. The primary property of chaos, which makes it useful in cryptography, is its sensitive dependence on initial conditions. The first application of a chaotically synchronized circuit was proposed by^22^. They linked two identical chaotic systems using standard signals and employed this concept to construct a chaotic synchronizing circuit. This idea further helped Baptista in the encryption of messages. Baptista-type algorithms are one of the chaos-based cryptographic algorithms among many proposed chaotic cryptosystems^6,23^. A comparative analysis of key schemes and improvements based on Baptista’s original approach is summarized in Table 1.

**Table 1:** Comparative analysis of baptista’s chaos-based cryptosystem.

References	Model	Pros	Cons	Finding
^2^	Superior iteration with extended logistic range	Stabilizes chaotic behavior	More computation per iteration	Improves logistic map behavior through two-step feedback
^3^	Modified Baptista using a square invertible matrix key	Better security against a one-time pad attack	Matrix inversion may increase cost	Protects against OTP and improves security robustness
^4^	Improved pseudo- random generation	Reduced vulnerability; enhanced randomness	No solution for the speed issue	Strengthens randomness using larger pseudo- random numbers
^5^	Security analysis of Baptista’s model	Identified weaknesses: critical evaluation	No new method proposed	Revealed vulnerability from X_0_ and high computational cost
^6^	Coupled 1D chaotic maps with the Baptista model	Increases unpredictability	Needs synchronization for coupled maps	Enhances encryption strength through coupling
^7^	Hybrid map system: logistic + tent + cubic + exponential + sine	High complexity, enhanced confusion, and diffusion	Computational overhead increases	Enriches encryption complexity by combining multiple chaotic maps
^9^	Dynamic lookup table in the Baptista algorithm	Faster execution; mitigates the lookup time problem	Slight increase in complexity	Addressed speed limitation by replacing static lookup with a dynamic one
^12^	Modulo-2 sum of the Baptista output and the logistic map	Fixes the non- uniform ciphertext issue	Implementation overhead	Addresses statistical imbalance in output
^13^	The gray ordering number used for chaos orbit analysis	Improves attack impact understanding	Limited to analysis only	Aids in understanding periodicity and attack influence
^23^	Chaos-based cryptography using the logistic map and static lookup table	Simple model; uses sensitivity and ergodicity of chaos	Vulnerable due to fixed initial condition (X_0_); slow computation due to static lookup	More secure than non-chaotic models, but has significant limitations
^24^	Attack analysis on the original Baptista model	Comprehensive vulnerability testing (entropy, OTP)	No countermeasures offered	Confirms high susceptibility to various attacks
Proposed Work	Modified Baptista with Parrondo’s paradox & Superior logistic map	Increased key space, uniform chaos, improved security	More parameters to manage	Overcomes non- uniformity and single-attractor limitation; resists various attacks

## Preliminaries

### Baptista algorithm

The original Baptista algorithm utilizes one-dimensional chaotic logistic map *f*(*x*) = *r x* (1 - *x*), where *x* ∈ [0, 1], for encryption^23^. For some initial value of *X*_0_, the map can be iterated as (1):

1 $${X_{\text{n}}}=r{\text{ }}{X_{{\text{n}} - {\text{1}}}}\left( {{\text{1 }} - {X_{{\text{n}} - {\text{1}}}}} \right),{\text{ for}}n={\text{ 1}},{\text{ 2}},{\text{ 3}},{\text{ }}...$$

**Fig. 1:**
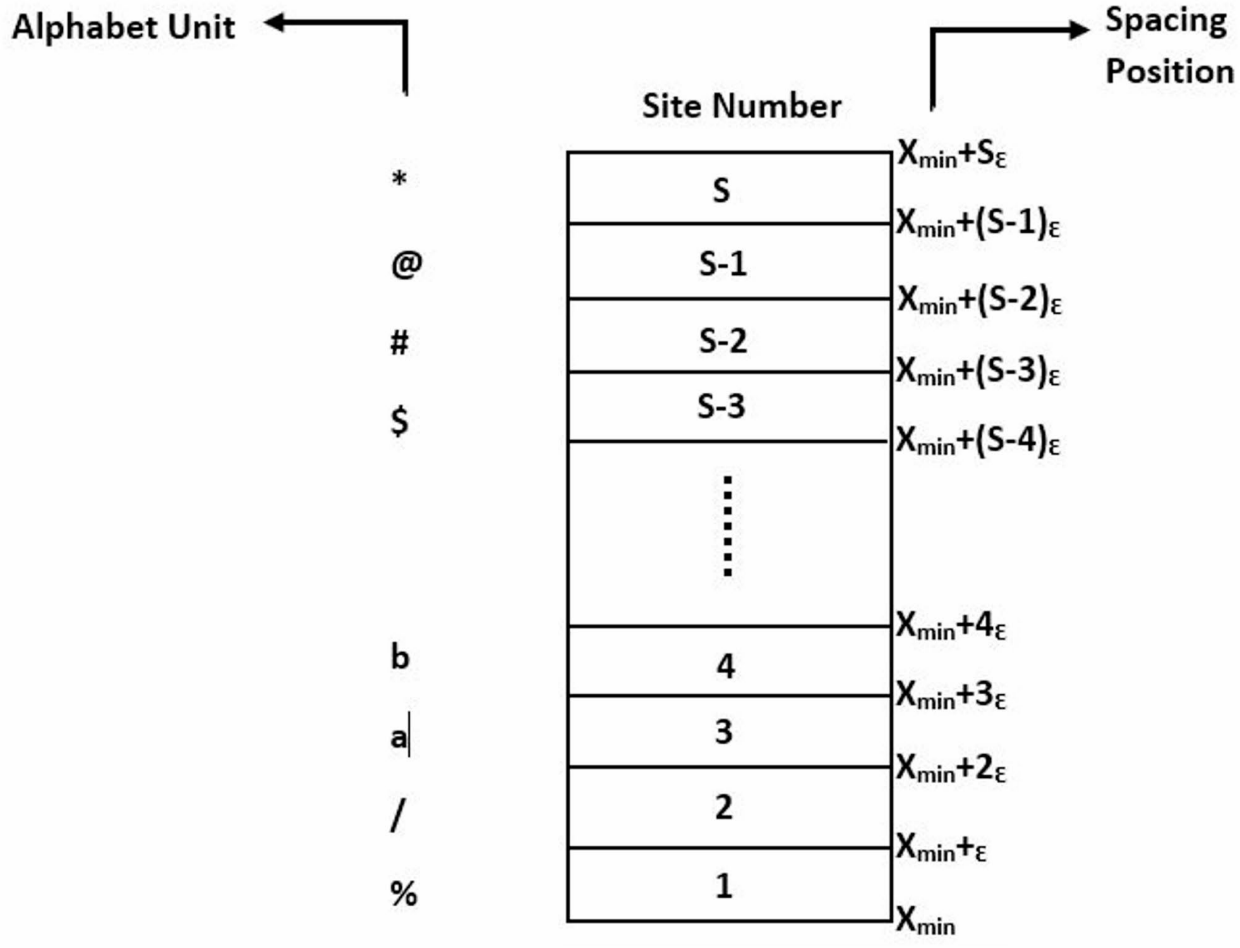
Scheme of representing an attractor with division into *S* sites, each site is of size ε = (*X*max - *X*min)/ *S*, where *X*min and *X*max cover some portion (or the whole) of the attractor.

Figure 1 shows the scheme of representing an attractor with division into *S* sites. Here, the size of each site is *ε =* (*Xmax - Xmin*)*/S*, where *Xmin* and *Xmax* encompass some portions of the attractor under consideration. Baptista chose *S* = 256 sites^18^. So, *S* (a finite set of 256 elements) is represented as *S* = {*S*_1_, *S*_2_, *S*_3_, ., *S*_i_, ., *S*_255_, *S*_256_}, where each *S*_*i*_ denotes the ASCII value of the corresponding character. The interval for each *S*_i_ = (*X*min + *S*_i−1_ε, *X*min + *S*_i_ε). Also, *X*min = 0.2 and *X*max = 0.8.

Let us consider that the plain text is *T* = {*T*_1_, *T*_2_, ., *T*_i_, .}, where each *T*_i_ represents the ASCII value of a character, and the corresponding Ciphertext is *C* = {*C*_1_, *C*_2_, ., *C*_i_, .}, where each *C*_i_ is a positive integer. Based on the above notations, the following is the original chaotic cryptographic scheme (Algorithm 1) due to Baptista^23^.

**Figure:**
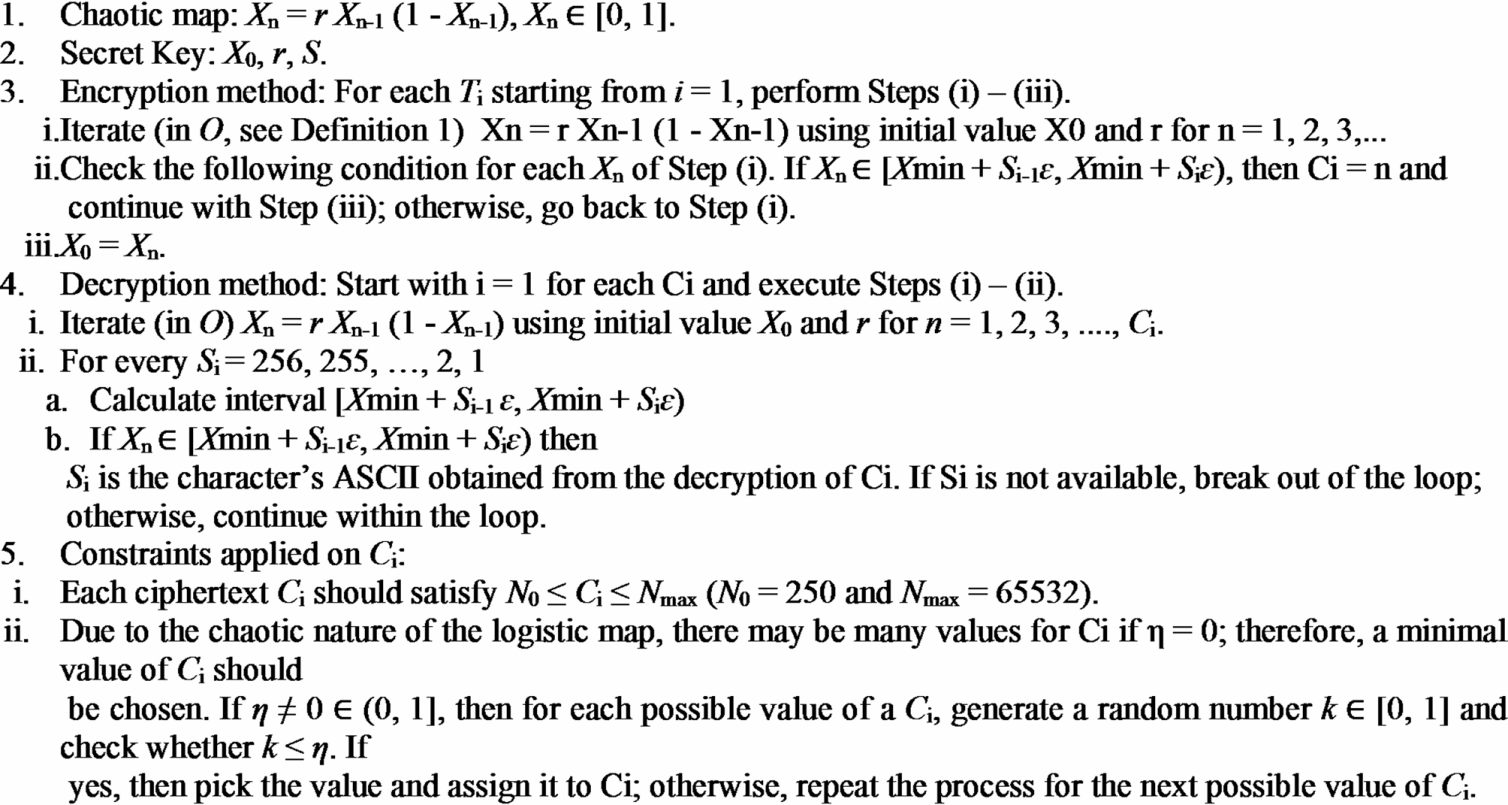
**Algorithm 1**. Baptista Algorithm.

### Parrondo’s paradox

Parrondo’s paradox is a counterintuitive concept originating from game theory, where two losing strategies, when combined in a specific alternating sequence, can result in an overall winning outcome. This phenomenon was first described by physicist Juan M.R. Parrondo in 1996 and is conceptually linked to the flashing Brownian ratchet in statistical mechanics^25^.

Consider two games:

- **Game A**: A biased coin toss that leads to an expected loss (e.g., win with probability 0.49, lose otherwise).
- **Game B**: A more complex game, often involving multiple biased coins, also results in a losing expectation when played individually.

Surprisingly, the expected outcome can be positive when Games A and B are played in a particular alternating sequence (e.g., A-B-B-A-B…). Further, even though $\:E\left[A\right]<0\:$and $\:E\left[B\right]<0$ hold true, $\:E\left[Alternation\right(A,B\left)\right]>0$ is also possible.

In the context of chaotic systems and cryptography, Parrondo’s paradox can be exploited by alternating between two ordered (non-chaotic) systems (e.g., two logistic maps with non-chaotic parameters) to produce chaotic dynamics, a behavior known as anti-control of chaos.

This mechanism enhances unpredictability in cryptographic schemes, as demonstrated in our Alternated Superior Baptista Algorithm, where we integrate two Superior logistic maps in an alternating manner. By leveraging Parrondo’s paradox in this way, the proposed method strengthens security through increased key complexity and improved randomness properties.

### Problems in Baptista’s algorithm

The Baptista algorithm, although foundational in chaos-based cryptography, exhibits several limitations that affect its practicality and security. One major drawback is the disproportionate length of the ciphertext compared to the plaintext, resulting in inefficient data transmission and storage. Additionally, the key space is relatively small, which makes the algorithm vulnerable to brute-force attacks. Security analyses have demonstrated that the Baptista algorithm is susceptible to various types of cryptanalytic attacks, including key recovery, entropy-based attacks, and weaknesses in the one-time pad. These vulnerabilities primarily arise from the deterministic nature of the underlying chaotic system. Another concern is performance. The encryption process requires a fixed number of iterations for convergence, making it significantly slower than conventional cryptosystems. This limitation is particularly critical when encrypting large multimedia files, where speed is essential. The reliance on a single chaotic attractor further reduces security, as it lacks the complexity and unpredictability that multiple attractors offer. Moreover, the non-uniform density function of the logistic map introduces statistical bias in the ciphertext, making it easier to analyze and break. Finally, the ciphertext distribution is often poor due to low iteration counts, resulting in patterns that reduce entropy and compromise confidentiality.

The following is a summary of various issues identified by^4,6,9,24^.

(i) The ciphertext is significantly longer than the plaintext and has a smaller key space.
(ii) It is insecure against key recovery, entropy, and one-time pad attacks.
(iii) The encryption process is slower than traditional ciphers due to the fixed number of iterations. Therefore, it is not ideal for securing large multimedia files.
(iv) A single chaotic attractor is less secure than multiple chaotic attractors.
(v) The non-uniformity of density functions in a single chaotic map makes it vulnerable.
(vi) The resultant ciphertext is obtained after a smaller number of iterations. Therefore, the distribution of ciphertext is not sufficiently secure.

#### Definition 1

**(Picard orbit)**: Let *X* be a non-empty subset of numbers and *f*: *X → X*. For an initial point *X*_0_ in *X*, the Picard orbit (generally called the orbit of *f*) is the set of all the iterates of a point *X*_0_, as shown in (2).

2 $$O\left( {f,{X_0}} \right):{\text{ }}={\text{ }}\{ {X_{\text{n}}}:{X_{\text{n}}}=~f({X_{\text{n}}}_{{ - {\text{1}}}}),n\,=\,{\text{1}},{\text{ 2}},{\text{ 3}}, \ldots \}$$

The orbit *O*(*f*, *X*_0_) of *f* at the point *X*_0_ is the sequence {*f*_n_(*X*_0_)}^2^.

#### Definition 2

**(Superior orbit)** Let *X* be a non-empty subset of numbers, and *f*: *X*→*X*. For an initial point *X*_0_, a sequence {*X*_n_} is constructed as described by (3).

3 $$\begin{gathered} {X_{\text{1}}}\,=\,{b_{\text{1}}}f\left( {{X_0}} \right){\text{ }}+{\text{ }}({\text{1}} - {b_{\text{1}}}){X_0}, \hfill \\ {X_{\text{2}}}\,=\,{b_{\text{2}}}f\left( {{X_{\text{1}}}} \right){\text{ }}+{\text{ }}({\text{1}} - {b_{\text{2}}}){X_{\text{1}}},~ \hfill \\ ~~~ \ldots \ldots \ldots \ldots \ldots \ldots \ldots \ldots \ldots \ldots ~~ \hfill \\ {X_{\text{n}}}\,=\,{b_{\text{n}}}f({X_{\text{n}}}_{{ - {\text{1}}}}){\text{ }}+{\text{ }}({\text{1}} - {b_{\text{n}}}){X_{\text{n}}}_{{ - {\text{1}}}}, \hfill \\ \end{gathered}$$

where 1 ≥ *β*_n_ > 0 and {*β*_n_} is convergent to a non-zero number.

The above-constructed sequence {*X*_n_} is a Superior sequence of iterates, denoted by *SO*(*f*,* X*_*0*_, *βn*), which reduces to *O*(*f*, *X*_0_) at *β*_n_ = 1^2,26^. The present work considers *β*_n_ = *β* for simplicity; however,^27^ provides a detailed study for reference.

#### Definition 3

(Superior Logistic Map): Iterating the logistic map in SO defines the Superior Logistic Map as follows (4).

4 $${X_{\text{n}}}=r{\text{ }}{X_{{\text{n}} - {\text{1}}}}\left( {{\text{1 }} - {X_{{\text{n}} - {\text{1}}}}} \right)\beta +{\text{ }}\left( {{\text{1 }} - \beta } \right){X_{{\text{n}} - {\text{1}}}},{X_{\text{n}}}~\left[ {0,{\text{ 1}}} \right]$$

Where *β*, *r*, and *X*_*n*_ are all real values, 0 < *β* ≤ 1.

Different forms of the logistic map have been integrated with Parrondo’s paradox in a deterministic and random manner^17,28–30^. According to Parrondo’s paradox, the two discrete dynamical systems are combined alternately while being iterated. The resulting dynamics may be cases of anti-control of chaos and control of chaos. The parameter switching (*PS*) algorithm, a generalized form of Parrondo’s paradox, has been applied to the Lorenz attractor, Rossler attractor, Chua attractor, etc., in a random and deterministic manner^31,32^.

#### Definition 4

**(Alternate Superior logistic map)**: Let us consider two Superior logistic maps L_1_ = *X*_n_ = *r*_1_ *X*_*n*−1_ (1 - *X*_*n*−1_) *β* + (1 - *β*) *X*_*n*−1_, and L_2_ = *X*_n_ = *r*_2_ *X*_*n*−1_ (1 - *X*_*n*−1_) *β* + (1 - *β*) *X*_*n*−1_, Iterating L_1_ and L_2_ alternatively defines the alternate Superior logistic map as follows.

L_1_L_2_ = *r*_1_ *X*_*n*−1_ (1 - *X*_*n*−1_) *β* + (1 - *β*) *X*_*n*−1_, when n is odd; and *r*_2_ *X*_*n*−1_ (1 - *X*_*n*−1_) *β* + (1 - *β*) *X*_*n*−1_, when n is even.

Classical Attack models comprise four different attack models^24,33^. These attack models describe the information available to the attacker when he attempts the attack. They can be categorized into four distinct groups.

- Ciphertext Only attack: The attacker possesses the ciphertext string.
- Known Plain text attack: The attacker possesses a series of plain text and corresponding ciphertext.
- Chosen Plain text attack: In this attack model, the attacker gets temporary access to the encryption machine. Thus, the attacker can choose any plaintext and get its corresponding ciphertext.
- Chosen Ciphertext attack: The attacker gets temporary access to the decryption machinery in this attack model. Thus, the attacker can choose any ciphertext and construct its plaintext.

In each of the above cases, the aim is to determine the used key, allowing the attacker to decrypt the targeted string.

## Proposed Baptista algorithm in superior orbit

### Superior Baptista algorithm

We implement the Baptista algorithm using the Superior logistic map. Here, *SO* adds parameter *β* to the secret key. Thus, the length of the key space increases. The logistic map produces chaotic data for a suitable choice of (*β*, *r*) ^2^. The complete algorithm, called the Superior Baptista Algorithm (Algorithm 2), is as follows.

**Figure:**
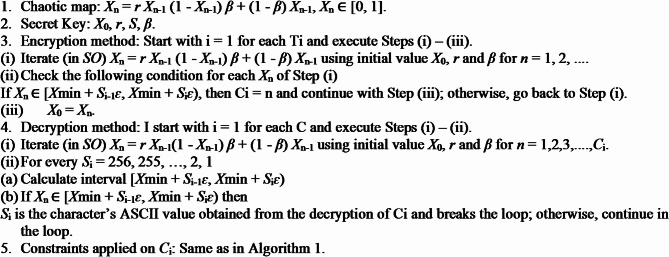
**Algorithm 2**. Superior Baptista Algorithm.

### Alternate superior Baptista algorithm

Now, we use the alternate Superior logistic map in the Baptista algorithm. There are certain instances of a triplet (*β*, *r*_1_, *r*_2_) for which order_1_ + order_2_ = chaos. Such instances of (*β*, *r*_1_, *r*_2_) may be helpful in the Baptista algorithm. In Algorithm 3, the key space further increases due to the inclusion of *r*_1_ and *r*_2_ in the secret key. The algorithm may be referred to as an alternating Superior Baptista Algorithm.

**Figure:**
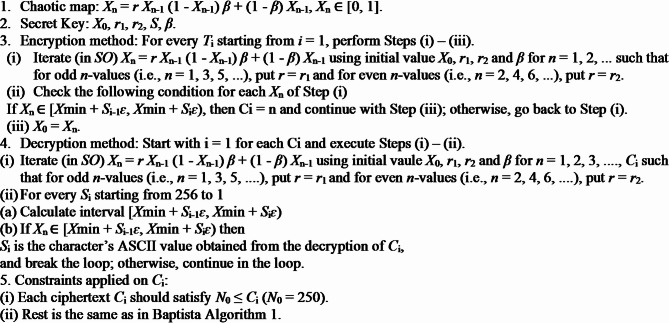
**Algorithm 3**. Alternated superior Baptista Algorithm.

**Table 2:** Comparative summary of Baptista algorithm variants.

Algorithm	Key parameters	Key space size	Encryption time	Chaotic behavior	Security strength
Baptista	*X*₀, *r*, *S*	Low	Fast	Moderate	Weak
Superior Baptista	*X*₀, *r*, *β*, *S*	Moderate	Moderate	High	Strong
Alternated Superior Baptista	*X*₀, *r*₁, *r*₂, *β*, *S*	High	Slower	Very High	Very Strong

A comparative overview of all three algorithms is provided in Table 2, highlighting the trade-offs in complexity, key space, encryption speed, and security. Furthermore, suppose *L* denotes the length of the message and *N_avg* represents the average number of iterations required to achieve the desired chaotic interval. In that case, the complexity of all the algorithms can be defined as O (*L* × *N_avg*).

***Examples***.

We take two examples to compare the security aspects of Algorithms 1, 2, and 3 based on the iteration numbers. In Algorithm 1, we choose *X*_0_ = 0.23232300000000 and *r* = 3.78 for the secret key as taken by^23^ for the sake of comparison. In Algorithm 2, we choose (*β*, *r*) = (0.9, 4.1) to produce a chaotic sequence. In both algorithms, *X*min and *X*max are chosen as 0.2 and 0.8, respectively^23^.

For Algorithm 3, an example of anti-control of chaos is picked up from the work of^34^, in which two ordered systems give rise to a chaotic system. They showed that for *r*_1_ = 4.76, *r*_2_ = 4.8034, and *β* = 0.7, there is a situation of order_1_ + order_2_ = chaos (anti-control of chaos)^26^. Here, we choose *X*min = 0.32 and *X*max = 0.98.

#### *Example 1*

Consider the encryption of a message “hello,” for example.

- *T* = {104, 101, 108, 108, 111} (ASCII values of “hello”).
- Intervals for the *T* are as follows:
  - For ‘h’ [0.44140625000000, 0.44375000000000),
  - for ‘e’ [0.43437500000000, 0.43671875000000),
  - for ‘l’ [0.45078125000000, 0.45312500000000),
  - and for ‘o’ [0.45781250000000, 0.46015625).

- Secret key:

- In Algorithm 1 (Baptista algorithm): *X*_0_ = 0.23232300000000, *r* = 3.78, *S* (ASCII-values set of all 256 characters).
- In Algorithm 2 (Superior Baptista Algorithm): *X*_0_ = 0.23232300000000, *r* = 4.1, *β* = 0.9, *S*.
- In Algorithm 3 (alternated Superior Baptista Algorithm): *X*_0_ = 0.23232300000000, *r*_1_ = 4.76, *r*_2_ = 4.8034, *β* = 0.7, *S*.

As shown in Table 3, encryption using the Superior Baptista Algorithm requires a higher number of iterations compared to encryption using the standard Baptista algorithm. Furthermore, the alternating Superior Baptista Algorithm requires the highest number of iterations for encryption.

**Table 3:** The ciphertext (number of iterations) for the plaintext “hello.”

Plaintext	ASCII-values of Plaintext (T)	Baptista algorithm	Superior Baptista Algorithm	Alternated Superior Baptista Algorithm
h	104	890	938	527
e	101	1095	1218	39,294
l	108	569	915	21,140
l	108	910	1313	17,984
o	111	285	649	97

#### *Example 2*

**Fig. 2:**
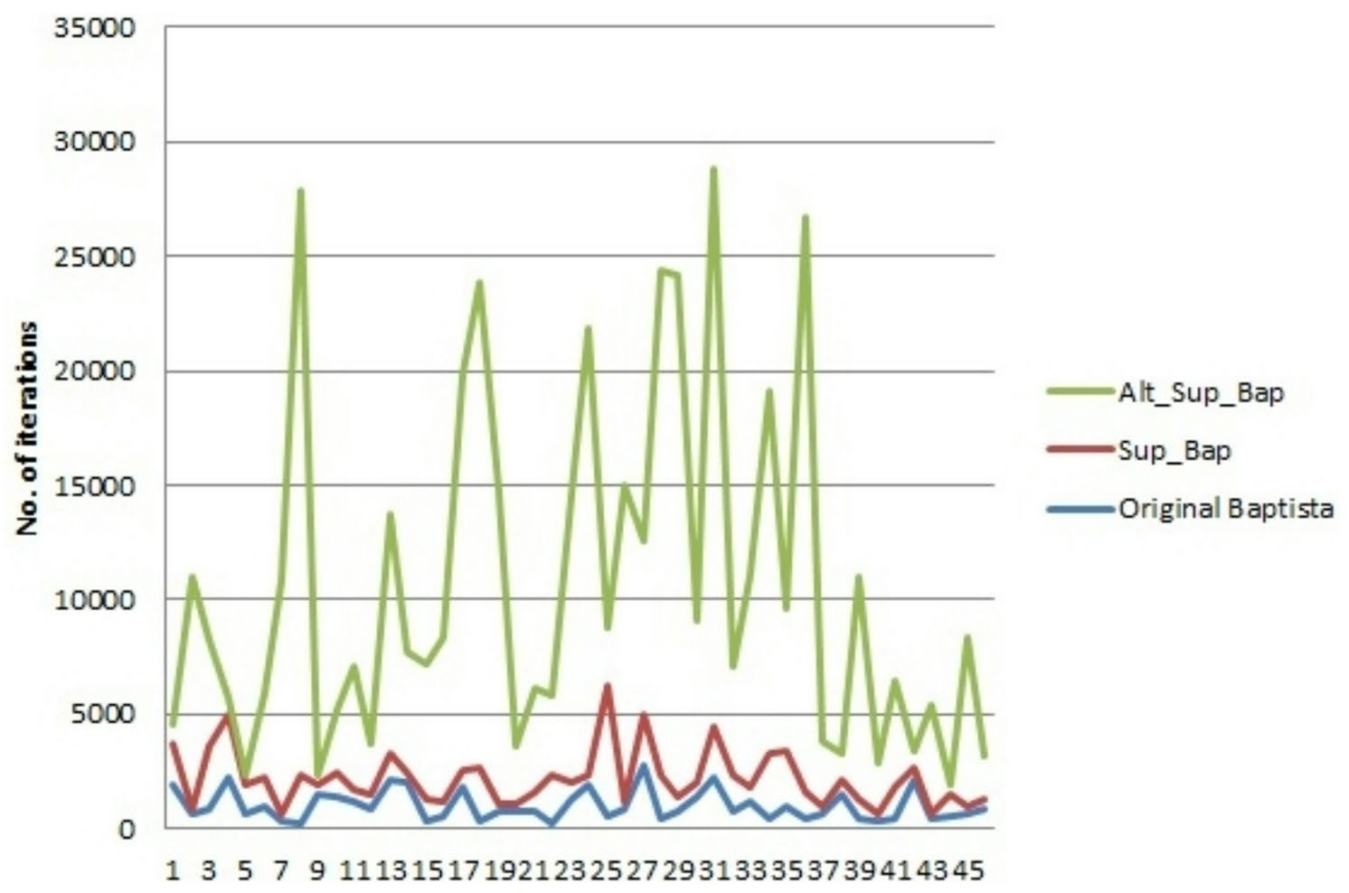
Comparison of several iterations for Baptista, Superior Baptista, and alternate Superior Baptista Algorithms.

Let us consider another example of encryption for the message “jaishreeramgyangunsagarjaikapistihulokujagarja,” containing 46 letters. We take the same initial condition and secret keys as in Example 1. Ciphertexts obtained from Baptista, Superior Baptista, and alternated Superior Baptista Algorithms for the input plaintext are given in Table 4. Figure 2 shows that the Superior Baptista Algorithm takes more iterations for encryption, and the alternate Superior Baptista Algorithm takes an extremely high number of iterations for encryption.

The alternate Superior logistic map is indeed an enhanced extension of the Superior logistic map, designed to increase the system’s chaotic behavior and key space by introducing an additional control parameter in the form of a second bifurcation value, $\:{r}_{2}$. While this results in a more complex and secure encryption process, it also significantly increases the computational overhead due to the alternating nature of parameter switching. Therefore, we first present the Superior Baptista Algorithm to show incremental improvement over the original Baptista approach, establishing the impact of introducing the Superior orbit (via parameter$\:\beta\:$) alone. Subsequently, the alternate Superior Baptista Algorithm is introduced as a further generalization that leverages anti-control of chaos and enhances key unpredictability.

**Table 4:** The ciphertext (number of iterations) for the plaintext “jaishreeramgyangunsagarjaikapistihulokujagarja”.

Plaintext	ASCII-values of Plaintext (T)	Baptista algorithm	Superior Baptista Algorithm	Alternated Superior Baptista Algorithm
j	106	1902	1839	1007
a	97	622	262	250
i	105	811	2817	30,766
s	115	2225	2721	14,992
h	104	643	1278	11,902
r	114	932	1278	23,682
e	101	362	279	45,070
e	101	197	2128	2376
r	114	1488	415	15,506
a	97	1364	1036	366
m	109	1178	530	40,452
g	103	830	701	31,226
y	121	2139	1094	1656
a	97	1981	431	796
n	110	324	914	18,922
g	103	514	661	22,808
u	117	1800	710	46,832
n	110	325	2315	44,724
s	115	716	312	32,282
a	97	799	301	264
g	103	737	873	32,498
a	97	269	2080	386
r	114	1266	699	31,072
j	106	1930	441	56,164
a	97	531	5709	324
i	105	905	272	86,556
k	107	2731	2211	35,140
a	97	477	1855	384
p	112	755	600	2328
i	105	1394	665	15,598
s	115	2197	2227	22,128
t	116	792	1498	21,600
i	105	1219	544	48,826
h	104	415	2823	3150
u	117	987	2422	2200
l	108	443	1139	3898
o	111	658	318	10,968
k	107	1508	640	538
u	117	393	876	20,760
j	106	315	277	22,244
a	97	390	1443	254
g	103	2073	540	14,034
a	97	388	266	448
r	114	505	944	12,658
j	106	604	325	4664
a	97	836	431	268

### Theoretical justification of the proposed enhancements

#### Security implications of superior orbits

In the standard Baptista algorithm, the logistic map is iterated as shown in Eq. (1), which constitutes the classical chaotic orbit. In the proposed Superior Baptista Algorithm, we utilize the Superior orbit as defined in (4). This convex combination induces a weighted influence of the prior state and the chaotic output, enhancing complexity and unpredictability. When *β* < 1, the resultant system introduces nonlinearity and memory, increasing the system’s sensitivity to initial conditions. Additionally, since the parameter *β* is now part of the secret key, the key space expands from (5):

5 $$Koriginal=\left\{ {X0,r} \right\}~to~Ksuperior=\left\{ {X0,r,\beta } \right\}$$

This increase in dimensionality directly enhances cryptographic strength, offering more robust resistance against key recovery and brute-force attacks. The Superior logistic map exhibits chaotic behavior over a broader range of parameters, enabling more flexible and secure configurations.

#### Parrondo’s paradox and the alternated superior logistic map

Parrondo’s paradox in dynamical systems suggests that alternating between two deterministic or regular (i.e., ordered) systems can result in globally chaotic dynamics. Inspired by this paradox, we define the Alternated Superior logistic map by alternating two Superior logistic maps *f*_*1*_ and *f*_*2*_ ​as follows (6) and (7):

6 $$Xn=r1.Xn - 1.\left( {1 - Xn - 1} \right)\beta +\left( {1 - \beta } \right).Xn - 1,~if~n~is~odd$$

7 $$Xn=r2 \cdot Xn - 1 \cdot \left( {1 - Xn - 1} \right)\beta +\left( {1 - \beta } \right) \cdot Xn - 1,~if~n~is~even$$

​Here, *r*_1_ and *r*_2_ are chosen such that each map may be near the edge of chaotic behavior, but their combination results in increased unpredictability, a case of anti-control of chaos. This strategy aligns with Parrondo’s principle, wherein “order + order = chaos.

## Results and discussions

The Baptista algorithm had specific problems, which have been addressed one by one.

**Larger key space**: The Superior Baptista Algorithm has a more expansive key space due to the addition of the *β* parameter to the secret key. The alternative Superior Baptista Algorithm allows the use of two chaotic attractors. Table 3 illustrates the ciphertext in terms of iteration counts required to encrypt the plaintext “hello” using the original Baptista algorithm, the Superior Baptista Algorithm, and the Alternated Superior Baptista Algorithm. Each character of the plaintext is first converted into its corresponding ASCII value, which serves as the target for the chaotic map during encryption. The original Baptista algorithm produces relatively moderate iteration counts for each character, such as 104 for ‘h’ and 111 for ‘o’. The Superior Baptista Algorithm, which introduces an additional secret parameter (*β*) to expand the key space, typically yields higher iteration counts, indicating increased complexity and enhanced security. Notably, the Alternated Superior Baptista Algorithm, which uses two chaotic attractors instead of one, yields significantly varied and often much higher iteration values, for instance, 101 for ‘e’ and 108 for ‘l’. This indicates a higher level of diffusion and randomness, enhancing resistance to statistical analysis and cryptographic attacks. Overall, the comparison demonstrates that the proposed enhanced algorithms improve the unpredictability and cryptographic strength of the original scheme by leveraging additional parameters and dual chaotic dynamics.

The Alternated Superior Baptista Algorithm, which alternates between two distinct chaotic attractors (e.g., different logistic map parameters), exhibits the most pronounced variability and encryption strength. This is evident in several instances, such as the character ‘i’ again appearing with an iteration count of 86556, or ‘u’ (ASCII 117) producing 46,832 and 20,760 iterations at different positions. These large and non-linear ciphertext values underscore the high sensitivity to initial conditions and character positions, core properties of chaos theory that enhance confusion and diffusion in encryption. Furthermore, the table illustrates how the same character can yield different ciphertexts in different positions within the plaintext. This reflects the dynamic nature of the chaotic process and the key evolution of the characters. For instance, the character ‘a’ appears multiple times with widely varying ciphertexts under each algorithm: 622, 1364, 1981, 799, 269, 531, 477, etc. in the original Baptista algorithm, each distinct due to the chaotic dependency on previous states.

**Use of multiple chaotic attractors**: Alternate Superior Baptista Algorithm uses two chaotic attractors: *X*_n_ = *r*_1_ *X*_*n*−1_ (1- *X*_*n*−1_) *β* + (1 - *β*) *X*_*n*−1_ and *X*_n_ = *r*_2_ *X*_*n*−1_ (1 - *X*_*n*−1_) *β* + (1 - *β*) *X*_*n*−1_, *X*_n_ $\:\in\:$ [0, 1]. Although the use of both chaotic attractors in the alternate Superior Baptista Algorithm does not produce a uniform distribution of chaotic data, it does result in a more secure encryption. However, the algorithm requires an extremely high number of iterations for encryption, which is a drawback. It can be seen from Table 4 that, in some cases, the number of iterations exceeds the maximum limit of 65,532 (specifically for the 26th plaintext).

**Fig. 3:**
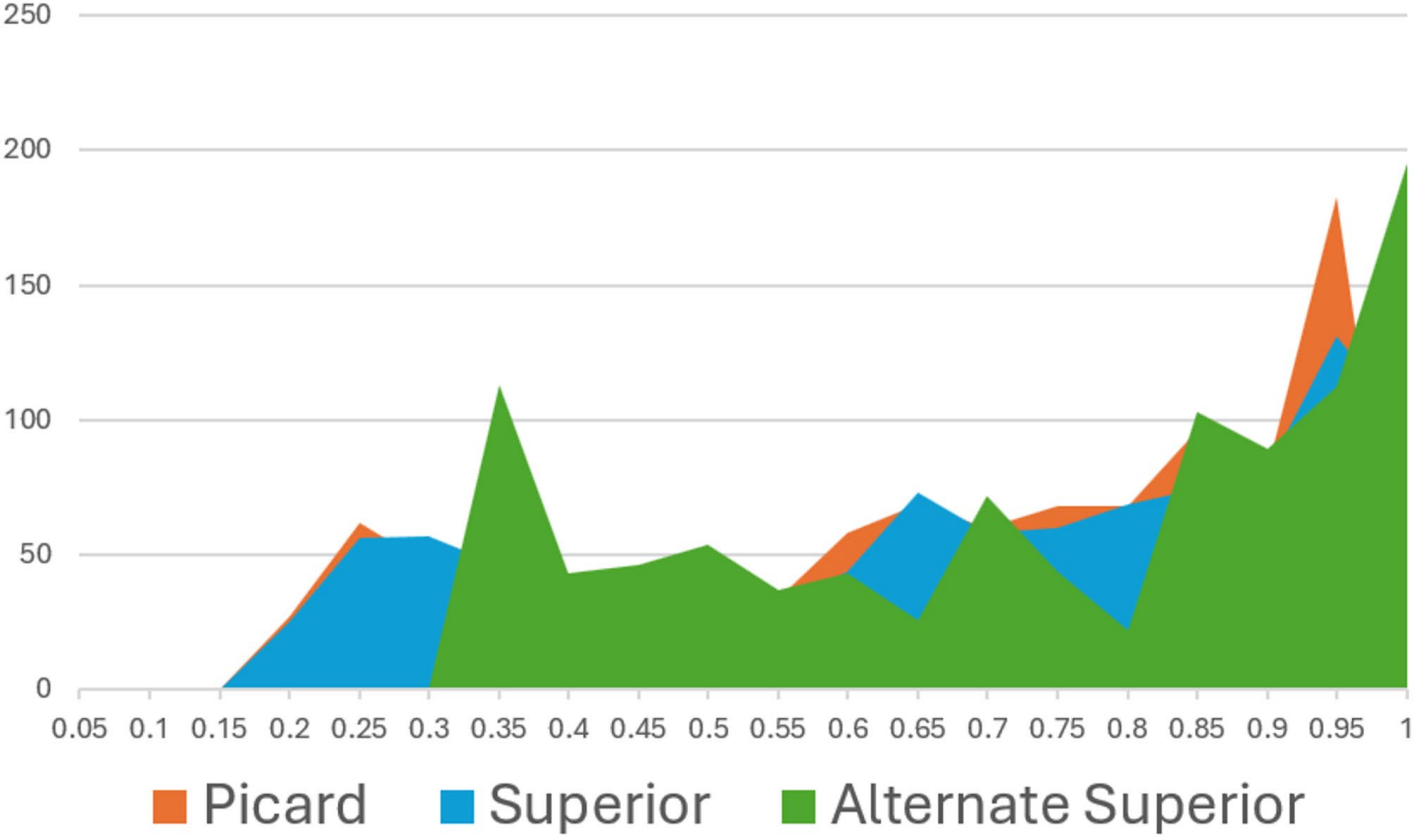
Distribution of chaotic values in the logistic map in different cases.

**High number of iterations**: Encryption should take a significant number of iterations to make the resultant ciphertext more secure. The Superior Baptista Algorithm takes a higher number of iterations, and the alternate Superior Baptista Algorithm takes an extremely high number of iterations.

**Encryption of large multimedia files**: A larger number of iterations increases the encryption process’s complexity. Though this is good for security, the process is slower. None of the algorithms is suitable for securing large multimedia files. *r*_2_, along with *β*, are included in the secret key.

**Uniform distribution of chaotic data**: Let us examine the values of the secret keys used in our examples. In the Superior Baptista Algorithm, *r* = 4.1 and *β* = 0.9 are in the secret key. The alternate Superior Baptista Algorithm uses *r*_1_ = 4.76, *r*_2_ = 4.8034, and *β* = 0.7 in the secret key. The logistic map in Picard orbit produces chaotic data for *r* = 3.78. Refer to Fig. 3 to see the distribution of chaotic values in all three cases. The logistic map in *SO* has a more uniform distribution of chaotic values than that of the Picard orbit. Due to more uniformly distributed chaotic data, a Superior logistic map takes more iterations for encryption.

The alternate Superior logistic map is less uniform than the logistic map in the Picard orbit. An interesting observation here is that the chaotic data is in the inter-interval (0.3, 0.1), unlike other cases of logistic maps. Therefore, we choose *X*_*min*_ = 0.32 and *X*_*max*_ = 0.98 in the alternate Superior Baptista Algorithm.

## The vulnerability analysis

The Baptista algorithm struggles against one-time pad attacks, entropy attacks, and key recovery attacks^35^. In all the attacks, there is a general assumption that the cryptanalyst knows everything about the cryptosystem, i.e., its design and operation, except for the secret key^24^. Here, our primary goal is to obtain the encryption key. To simplify analysis, we use symbols of the form 2^i^, where *i* = 1, 2, 3, ., 8. Symbols are nothing but plain text. Here we assume that in Baptista-type algorithms, *N*_0_ = 0, *η* = 0, and the source emits two symbols *S*_2_ = {*s*_1_, *s*_2_}. The secret keys in algorithms (Algorithm 1, Algorithm 2, and Algorithm 3) are used as given by ^23,35^. Secret keys are (*X*_*0*_, *r*,* S*) = (0.232323, 3.78, 256), (*X*_*0*_, *r*,* S*,* β)* = (0.232323, 4.1, 256, 0.9), (*X*_*0*_, *r*_*1*_, *r*_*2*_, *S*,* β)* = (0.232323, 4.76, 4.8034, 256, 0.7) in Baptista, Superior Baptista and alternate Superior Baptista Algorithms respectively. Also, in Algorithms 1 and 2 (*X*_*min*_, *X*_*max*_*)* = (0.2, 0.8) and Algorithm 3 (*X*_*min*_, *X*_*max*_) = (0.32, 0.98). The range is different in Algorithm 3 due to the nature of chaotic attractors^35^.

### One-time pad attack

The one-time pad was an encryption scheme^33^. Let’s understand the attack with an example. For a set of two symbols, source *S* = {*s*_*1*_, *s*_*2*_}. In Algorithms 1 and 2, ε = (0.8–0.2) / 2 = 0.3 (see Fig. 1). So, symbol *s*_1_ is in the interval [0.2, 0.5], and symbol *s*_2_ is in the interval [0.5, 0.8]. While in Algorithm 3, ε = (0.98 − 0.32) / 2 = 0.33. So the symbol *s*_1_ is in the interval [0.32, 0.65] and symbol *s*_2_ is in the interval [0.65, 0.98].

In the chosen plain text attack, we apply Baptista-type algorithms on plain texts *P*_*1*_ = (*s*_1_*s*_1_*s*_1_ *s*_1_*s*_1_*s*_1_*s*_1_*s*_1_*s*_1_*s*_1_…) and *P*_*2*_ = (*s*_2_*s*_2_*s*_2_*s*_2_*s*_2_*s*_2_*s*_2_*s*_*2*_*s*_2_*s*_2_…). Thus when we apply Baptista Algorithm on plain texts, we get ciphertexts *C1* = (5, 3, 2, 2, 2, 3, 2, 3, 2, 2, …) and *C2* = (1, 2, 3, 9, 5, 7, 5, 1, 1, 1, .) respectively. The cumulative sum of *C1* = {5, 8, 10, 12, 14, 17, 19, 22, 24, 26, …} and *C2* = {1, 3, 6, 15, 20, 27, 32, 33, 34, 35, …}. So the one-time pad is *O* **=** *xs*_2_*xs*_2_*xs*_1_*s*_2_*xs*_1_*xs*_1_*xs*_1_*xs*_1_*s*_2_*xs*_1_*xs*_1_*s*_2_*xs*_1_*xs*_1_*xs*_1_*s*_2_…., where the first *x* represents *x*_0_ and the rest *x* are outside the interval [0.2, 0.8]. This is the one-time pad, which is equivalent to the key. Now, any ciphertext can be decrypted. For example, if C = (3, 5, 2, 2, 8), *P* would be (*s*_2_*s*_1_*s*_1_*s*_1_*s*_2_).

Similarly, in case of Superior Baptista Algorithm, *C1* = (8, 6, 2, 3, 4, 3, 2, 8, 6, 5 ) and *C2* = ( 1, 2, 3, 3, 1, 1, 1, 3, 4, 3 ), and the cumulative sum of *C1* = {8, 14, 16, 19, 23, 26, 28, 36, 42, 49,…} and *C2* = {1, 3, 6, 9, 10, 11, 12, 17, 21, 24,…}. So, the one-time pad.

$$O\,=\,x{s_{\text{2}}}x{s_{\text{2}}}xx{s_{\text{2}}}x{s_{\text{1}}}{s_{\text{2}}}{s_{\text{2}}}{s_{\text{2}}}{s_{\text{2}}}x{s_{\text{1}}}x{s_{\text{1}}}{s_{\text{2}}}x{s_{\text{1}}}x{s_{\text{2}}}x{s_{\text{1}}}{s_{\text{2}}}x{s_{\text{1}}}.....$$

In case of alternate Superior Baptista Algorithm, *C1* = (3, 2, 2, 2, 4, 2, 2, 2, 2, 2 ) and *C2* = (1, 1, 2, 2, 4, 1, 1, 2, 2, 4 ). Cumulative sum of *C1* = {3, 5, 7, 9, 13, 15, 17, 19, 21, 23,…} and *C2* = {1, 2, 4, 6, 10, 11, 12, 14, 16, 20,…}. So, the one-time pad.

$$O\,=\,x{s_{\text{2}}}{s_{\text{2}}}{s_{\text{1}}}{s_{\text{2}}}{s_{\text{1}}}{s_{\text{2}}}{s_{\text{1}}}x{s_{\text{1}}}{s_{\text{2}}}{s_{\text{2}}}{s_{\text{2}}}{s_{\text{1}}}{s_{\text{2}}}{s_{\text{1}}}{s_{\text{2}}}{s_{\text{1}}}x{s_{\text{1}}}{s_{\text{2}}}{s_{\text{1}}}{s_{\text{2}}}{s_{\text{1}}}{s_{\text{2}}}....$$

Similarly, for the four symbols *S* = {*s*_1_, *s*_2_, *s*_3_, *s*_4_}, the parameter ε is 0.15 for Algorithms 1 and 2 and 0.165 for Algorithm 3. The symbols s_1_, s_2_, s_3_, and s_4_ have sites [0.2, 0.35], [0.35, 0.5], [0.5, 0.65], and [0.65, 0.8], respectively, for Algorithms 1 and 2. The symbols *s*_1_, *s*_2_, *s*_3_, and *s*_4_ have sites [0.32, 0.485], [0.485, 0.65], [0.65, 0.815], and [0.815, 0.98], respectively, for Algorithm 3. Table 5 shows the results when Algorithm 1, Algorithm 2, and Algorithm 3 are applied to the plaintexts *P*1 = (*s*_1_*s*_1_*s*_1_*s*_1_*s*_1_*s*_1_*s*_1_*s*_1_…), *P*2 = (*s*_2_*s*_2_*s*_2_*s*_2_*s*_2_*s*_2_*s*_2_*s*_2_…), *P*3 = (*s*_3_*s*_3_*s*_3_*s*_3_*s*_3_*s*_3_*s*_3_*s*_3_…), and *P*4 = (*s*_4_*s*_4_*s*_4_*s*_4_*s*_4_*s*_4_*s*_4_*s*_4_…) to get the corresponding ciphertexts and one-time pad.

**Table 5:** Results of algorithm 1, algorithm 2, and algorithm 3 on plaintexts *P*1, *P*2, *P*3, and *P*4.

	Baptista Algorithm	Superior Baptista Algorithm	Alternate Superior Baptista Algorithm
Ciphertext *C1*	5, 9, 5, 3, 4, 5, 10, 13, 13, 4, …	8, 8, 3, 4, 5, 8, 6, 7, 6, 18, …	3, 2, 4, 4, 2, 4, 4, 2, …
Ciphertext *C2*	8, 2, 2, 5, 7, 5, 10, 20, 27, 5…	14, 12, 42, 9, 19, 2, 5, 10, 6 12…	7, 10, 4, 8, 6, 6, 6, 6, …
Ciphertext *C3*	3, 3, 14, 7, 10, 15, 5, 6, 3, 4, …	3, 3, 11, 4, 10, 3, 6, 4, 3, 6, …	1, 10, 22, 6, 6, 6, 6, 6, …
Ciphertext *C4*	1, 14, 17, 1, 1, 1, 1, 6, 1, 1, …	1, 8, 1, 1, 1, 12, 5, 8, 13, 1, …	2, 2, 2, 4, 2, 2, 2, 4, …
Cumulative sum of *C1*	5, 14, 19, 22, 26, 31, 41, 54, 61, 65, …	8, 16, 19, 23, 28, 36, 42, 49, 55, 73, …	3, 5, 9, 13, 15, 19, 23, 25, 27, 31, …
Cumulative sum of *C2*	8, 10, 12, 17, 24, 29, 39, 59, 86, 91, …	14, 26, 68, 77, 96, 98, 103, 113, 119, 131 …	7, 17, 21, 29, 35, 41, 47, 53, …
Cumulative sum of *C3*	3, 6, 20, 27, 37, 52, 57, 63, 66, 70, …	3, 6, 17, 21, 31, 34, 40, 44, 47, 53, …	1, 11, 33, 39, 45, 51, 57, 63, …
Cumulative sum of *C4*	1, 15, 32, 33, 34, 35, 36, 42, 43, 44, …	1, 9, 10, 11, 12, 24, 29, 37, 38, 50, 51, …	2, 4, 6, 10, 12, 14, 16, 20, 22, 24, …
One-time pad *O*	*xs*_4_*xs*_3_*xs*_1_*s*_3_*xs*_2_*xs*_2_*xxxs*_1_*s*_4_*xs*_2_*xs*_1_…	*xs*_4_*xs*_3_*xxs*_3_*xs*_1_*s*_4_*s*_4_*s*_4_*s*_4_*xs*_2_*xs*_1_*s*_3_*xs*_*1*_.	*xs*_3_*s*_4_*s*_*1*_*s*_4_*s*_1_*s*_4_*s*_2_*xs*_1_*s*_4_*s*_3_*s*_4_*s*_1_*s*_4_*s*_1_*s*_4_*s*_2_*xs*_1_*s*_4_ *…*

We can observe that the cryptanalyst can easily construct a one-time pad in all three algorithms. This is because each symbol is associated with a different site, and each site corresponds to some non-overlapped intervals in Baptista-type algorithms. Thus, there will be no overlapping of *X*_*n*_ values. Hence, non-overlapped ciphertexts are obtained.

### Entropy attack

Entropy is a mathematical measurement of information or uncertainty. It is computed as shown in Eq. (8).

8 $${\text{H}}\left( {\text{S}} \right){\text{ }}=\sum {\text{P}}({s_i}){\text{log }}\left( {{\text{ 1}}/{\text{P}}\left( {{s_i}} \right){\text{ }}} \right){\text{ bits}}$$

Here, P(*s*_*i*_) denotes the probability of the symbol *s*_*i*_, which can be calculated as a function of the probability distribution. The entropy should be 1 when encryption is applied to two inputs. Let us take an example of tossing a coin; the entropy will be one because, in one bit, we can encrypt it as 0 for a head and 1 for a tail^33^. If entropy is less than 1, it may lead to some security threats. In this attack, we associate each value with a particular range. In the case of two symbols, the range of *X*_n_ is [0.2, 0.5] and [0.5, 0.8] (for Algorithm 1 and Algorithm 2), and [0.32, 0.65] & [0.65, 0.98] (for Algorithm 3). The first symbol, *s*_1_, *is associated with the range [0.2*,* 0.5]*,* and s*_2_ *has a* range [0.5, 0.8] (for Algorithm 1 and Algorithm 2). While the symbol *s*_1_ is associated with the range [0.32, 0.65], *s*_2_ has a range [0.65, 0.98] (for Algorithm 3). We input a random message of 1000 length with *s*_1_ and *s*_2_ and iterate them individually. We record the number of times each symbol appears at every iteration. Based on this calculation, the symbol with maximum frequency is set as the most probable symbol at a given iteration number.

This is a ciphertext-only attack. In^24^, with the same *r* and *x*_0_ values, the Baptista algorithm can decrypt 70% of symbols, and the entropy value is much less than 1. The formula for computing the entropy of the encryption process is given by (9).

9 $$H\left( {{C_{S2}}} \right)=\mathop \sum \limits_{{i=1}}^{N} P\left( i \right)\mathop \sum \limits_{{j=1}}^{{S2}} S_{j}^{{\left( i \right)}}\log \left( {\frac{1}{{P\left( {S_{j}^{{\left( i \right)}}} \right)}}} \right)$$

Where *i* is the number of iterations used to encrypt the symbol, *N* shows the maximum iteration, $S_{j}^{{\left( i \right)}}$ represents the symbol, and *j* = {1, 2} (in case of 2 symbols).

From Table 6, the following observations are made.

1. The first symbol is *s*_2_ because the *s*_1_ value is zero in the Baptista and Superior Baptista Algorithms. However, this is not the case with the Alternate Superior Algorithm. In the case of the Baptista encryption scheme, the *s*_1_ value becomes zero three times while the *s*_2_ value is non-zero, i.e., 100% of the *s*_2_ value in those instances, which is prone to attack. A similar case was once seen in the Superior Baptista Algorithm. Alternate Superior Baptista encryption has no such case. However, some unpredictability is inevitable since the input sequence is not fixed.
2. In the Baptista encryption scheme, the *s*_2_ value becomes zero thrice while the *s*_1_ value is non-zero, i.e., 100% of *s*_1_ is at instances prone to attack. A similar case occurs twice and thrice in Superior Baptista encryption and alternate Superior Baptista encryption schemes, respectively. However, the cases are not fixed due to the random input sequence, thus keeping the scope of unpredictability.
3. Further, there are instances where both *s*_1_ and *s*_2_ are zero, which means that at that particular iteration, the functional value is in [0, 0.2] or [0.8, 1] in the case of Baptista and alternate Superior algorithms and [0, 0.32] or [0.98, 1] in case of alternate Superior Baptista Algorithm. In the Baptista algorithm, the symbols *s*_1_ and *s*_2_ became zero at two instances. Such a situation arises once and 7 times in Superior Baptista and alternate Superior Baptista encryption methods, respectively.
4. Thus, in all three algorithms, the alternate Superior Baptista Algorithm showed unusual behavior compared to the other two, which makes it less prone to attack and more secure.

**Table 6:** Comparison of Baptista, superior Baptista, and alternate superior Baptista algorithms for *s*_1_ and *s*_2_ values (symbol 1 and symbol 2) after each iteration.

Iteration	In the case of Baptista algorithms		In the case of the Superior Baptista Algorithm		In the case of the Alternate Superior Baptista Algorithm	
	*s*_1_ values	*s*_2_ values	*s*_1_ values	*s*_2_ values	*s*_1_ values	*s*_2_ values
01	00	73	00	203	129	274
02	377	24	187	75	212	75
03	62	132	43	82	59	102
04	34	36	76	42	29	42
05	21	122	71	41	18	00
06	05	00	42	17	22	00
07	04	45	38	23	00	00
08	01	00	27	01	38	00
09	00	13	10	04	00	00
10	00	00	04	00	00	00
11	02	16	04	02	00	00
12	01	00	00	00	00	00
13	00	32	01	02	00	00
14	00	00	03	00	00	00

Table 7 is the result of the frequency analysis of symbols by the Baptista algorithm. At the first iteration, the symbol *s*_1_ is zero, leading to the predictability of the very first symbol as *s*_2_. At iterations 10 and 14, the frequencies of *s*_1_ and *s*_2_ are zero. The entropy calculated here using Eq. (3) is 0.49, nearly half the ideal value of 1. There are cases where either of the frequencies is zero, and the other is not. This means the guessing of the non-zero frequency symbol is 100% correct. The actual frequency analysis depends on random inputs.

**Table 7:** After each iteration, the total frequency of *s*_1_ and *s*_2_ representing the highest value will be the most probable symbol for a given number of iterations s* by the Baptista Algorithm.

Iterate	Frequency	s_1_(Frequency)	s_2_(Frequency)	s*
1	0.073	0.00	1.00	73(*s*_2_)
2	0.401	0.94	0.06	377(*s*_1_)
3	0.194	0.32	0.68	132(*s*_2_)
4	0.070	0.49	0.51	36(*s*_2_)
5	0.143	0.15	0.85	122(*s*_2_)
6	0.005	1.00	0.00	5(*s*_1_)
7	0.049	0.08	0.92	45(*s*_2_)
8	0.001	1.00	0.00	1(*s*_1_)
9	0.013	0.00	1.00	13(*s*_2_)
10	0.000	0.00	0.00	----
11	0.018	0.11	0.89	16(*s*_2_)
12	0.001	1.00	0.00	1(*s*_1_)
13	0.032	0.00	1.00	32(*s*_2_)
14	0.000	0.00	0.00	----

In Table 8, like Table 7, we see that the first symbol is *s*_2_ because *s*_1_ is zero. This is predictable. The entropy calculated here is 0.69 on the same random input. This indicates that the Superior Baptista Algorithm is more robust than the Baptista algorithm, as it exhibits greater unpredictability compared to the Baptista algorithm.

**Table 8:** The total frequency of *s*_1_ and *s*_2_ after each iteration, representing the highest value, will be the most probable symbol for a given number of iterations s* by the superior Baptista Algorithm.

Iterate	Frequency	s_1_(Frequency)	s_2_(Frequency)	s*
1	0.203	0.00	1.0	203(*s*_2_)
2	0.262	0.71	0.29	187(*s*_1_)
3	0125	0.34	0.66	82(*s*_2_)
4	0.118	0.64	0.36	76(*s*_1_)
5	0112	0.63	0.37	71(*s*_1_)
6	0059	0.71	0.29	42(*s*_1_)
7	0.061	0.62	0.38	38(*s*_1_)
8	0.028	0.96	0.04	27(*s*_1_)
9	0.014	0.71	0.29	10(*s*_1_)
10	0.004	1.00	0.00	04(*s*_1_)
11	0.006	0.67	0.33	04(*s*_1_)
12	0.000	0.00	0.00	----
13	0.003	0.33	0.67	02(*s*_2_)
14	0.003	1.00	0.00	03(*s*_1_)

Table 9 indicates that unpredictability is very high, as the data contains a high proportion of zeros. At the first iteration, *s*_1_ is non-zero, unlike the other two algorithms. The calculated entropy is 0.83 for the same random input, closer to the ideal value. However, all these frequencies depend on the values we have taken and the number of iterations we performed. To be sure, we consider another example of a string of 1,000 input symbols, consisting of the set *s*_2_. Again, we found that the Baptista algorithm is vulnerable to an entropy attack, as the entropy calculated is 0.53. The entropy calculated for the Superior and Alternate Superior Algorithms is 0.70 and 0.82, respectively. All the characteristics described above are the same.

**Table 9:** The total frequency of *s*_1_ and *s*_2_ after each iteration, representing the highest value, will be the most probable symbol for a given number of iterations, s*, by the alternate superior Baptista Algorithm.

Iterate	Frequency	s_1_(Frequency)	s_2_(Frequency)	s*
1	0.403	0.3	0.7	274(*s*_2_)
2	0.287	0.7	0.3	212(*s*_1_)
3	0.161	0.4	0.6	102(*s*_2_)
4	0.71	0.4	0.6	42(*s*_2_)
5	0.018	1.00	0.00	18(*s*_1_)
6	0.022	1.00	0.00	*22(s*_1_)
7	0.00	0.00	0.00	----
8	0.038	1.00	0.00	38(*s*_1_)
9	0.000	0.00	0.00	----
10	0.000	0.00	0.00	----
11	0.000	0.00	0.00	----
12	0.000	0.00	0.00	----
13	0.000	0.00	0.00	----
14	0.000	0.00	0.00	----

Thus, the entropy attack will not succeed in Algorithms 2 and 3.

### Key recovery attacks

#### Initial value estimation

This cryptanalysis method is based on applying Gray codes, as shown in^15^. In this attack using gray codes, it is possible to estimate the initial *X*_0_ value. Since the chaotic values are sensitively dependent on initial values, revealing the *X*_0_ value could lead to the key and further the message. The attack uses (10) to obtain the Mandelbrot map.

10 $${X_{{\text{n}}+{\text{1}}}}={X_{\text{n}}}^{{\text{2}}}+c$$

**Fig. 4:**
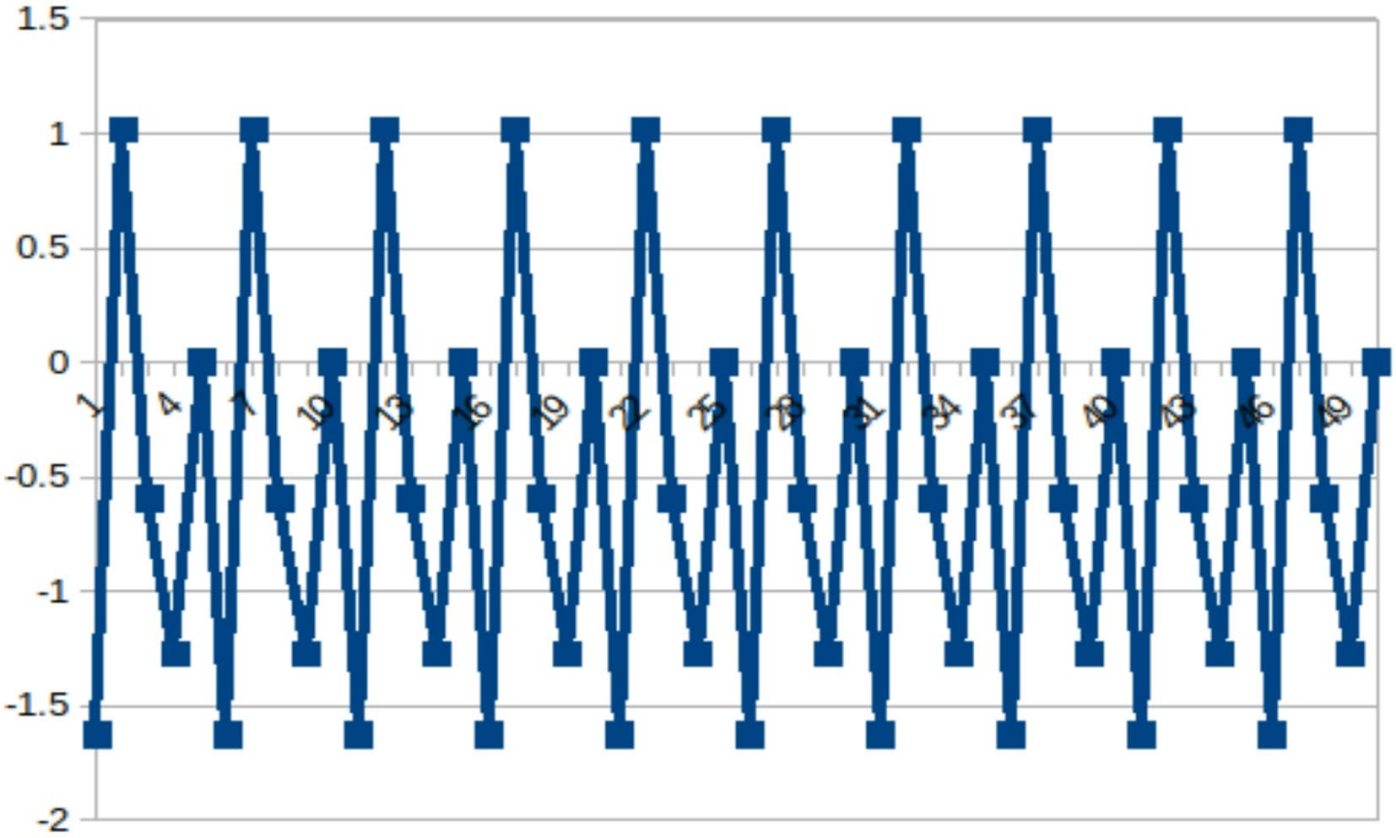
A 5-Periodic curve at *c* = -1.625413725, *X*_0_ = 0 and sequence *CLRLL*^13^.

The range of the variable *c* can be determined by |2/*β|*^35^. In the Baptista algorithm, the range of the c value is [-2, 2] as *β* = 1, while in the Superior algorithm where *β* = 0.9, the range is [-2.22, 2.22], and in the alternate Superior algorithm where *β* = 0.7, the range is [-2.85, 2.85]. In^13^, it is shown how 1D quadratic maps can be formulated using Gray codes. Here, the letters *L*, *R*, and *C* indicate whether each iteration falls to the left (*X*_*i*_ < 0), right (*X*_*i*_ > 0), or at the critical point (*X*_*i*_ = 0) of the map, respectively. Figure 4 shows an example of the Gray code obtained at *c* = -1.625413725 and *X*_0_ = 0. In the Attack, we associate the *s*_1_ symbol with the left region (*L*) and the *s*_2_ symbol with the correct region (*R*). So, the plain text containing two symbols will be iterated, and the orbit will be maintained based on the region obtained after each iteration. The number of iterations after which the expected region associated with the symbol occurs will become the cipher.

To get the value, due to *X*_0_, the orbit will be converted into gray code g_0_ by changing 0 to R and 1 to *L*. Add and subtract one in the original Gray code to get two more Gray codes, *g*_+ 1_ and *g*_− 1_. Now let’s take the standard part in (g_0_, *g*_*+* 1_) and (*g*_0_, *g*_− 1_) and then substitute zero by + and one by *-* and do the calculation on (*√-c*) as described in^14^. For example, 01 will be calculated as [*+ √(-c-√-c)*], and we get an upper limit and lower limit of *X*_*0*_.

We can equate the one-dimensional logistic map (1) with (7) by putting *c* = *r* * (2 - *r*) / 4^23^. The value *c* = -1.8 can be estimated to be within the range of [-2, 2]. This *c* value corresponds to *r* = 3.87. However, as in the paper, we have chosen *r* = 3.78 for the Baptista algorithm. Therefore, we choose the corresponding *c* value, which is -1.68. For the Superior algorithm at *β* = 0.9, the *c* value will be -2.1525, corresponding to *r* = 4.10. In the case of the alternate Superior algorithm, for *r_odd* = 4.76, the value of *c* is -3.2844, and for *r_even* = 4.8034, the value of *c* is -3.36646.

Now, let us understand the attack with an example of the input string *s*_1_*s*_2_*s*_2_*s*_1_*s*_1_*s*_1_*s*_1_, and here we set *X*_0_ = 0.232323 in all the following examples.

##### *Example 3*

As in^24^, *c* = − 1.8 (so *r* = 3.87), and the resultant value of a range of *X*_0_ is (0.2254207616, 0.243441280).

##### *Example 4*

In the Baptista algorithm, by using *r* = 3.78 and *c* = -1.68, the orbit array *O* is (*RLRLLLLRLRLRLL*), and the resultant cipher array *w* is (1, 1, 5, 1, 2, 2, 1). To obtain the narrow range of *X*_0_ values, we generate the Gray code of *O*, which is (0, 1, 0, 1, 1, 1, 1, 0, 1, 0, 1, 0, 1, 1). Further, we construct *g*_*+ 1*_ and *g*_*− 1*_. Thus, we get the following.

$${g_{ - \,{\text{1}}}}=0{\text{1}}0{\text{1111}}0{\text{1}}0{\text{1}}0{\text{1}}0$$

$${g_0}\,=\,0{\text{1}}0{\text{1111}}0{\text{1}}0{\text{1}}0{\text{11}}$$

$${g_{+\,{\text{1}}}}=0{\text{1}}0{\text{1111}}0{\text{1}}0{\text{1}}00{\text{1}}$$

The standard part in (*g*_0_, *g*_+ 1_) is 010111101010, and in (*g*_0_, *g*_− 1_) is 0101111010101. *T*he rest of the calculation on the standard part in (g_0_, g_+ 1_) by substituting one by – and zero by + is as follows:

*(-c-(-c* + *(-c-(-c-(-c-(-c-(-c* + *(-c-(-c* + *(-c-(-c* + *(-c)*^*0.5*^*)*^*0.5*^*)*^*0.5*^*)*^*0.5*^*)*^*0.5*^*)*^*0.5*^*)*^*0.5*^*)*^*0.5*^*)*^*0.5*^*)*^*0.5*^*)*^*0.5*^*)*^*0.5*^

Also, by substituting, 1 by – and 0 by + in common part in (*g*_*0*_, *g*_*− 1*_), we get.

*(-c-(-c* + *(-c-(-c-(-c-(-c-(-c* + *(-c-(-c* + *(-c-(-c* + *(-c-(-c)*^*0.5*^*)*^*0.5*^*)*^*0.5*^*)*^*0.5*^*)*^*0.5*^*)*^*0.5*^*)*^*0.5*^*)*^*0.5*^*)*^*0.5*^*)*^*0.5*^*)*^*0.5*^*)*^*0.5*^*)*^*0.5*^

Thus, the estimated value of *X*_*0*_ is (0.23207892, 0.23462428). By adjusting the value of *c*, the range of *X*_0_ becomes narrower. Now, the Superior algorithm is applied to the exact input string. The cipher array is (1, 1, 5, 1, 2, 1, 1), and the orbit array is *RLRLLLLRLRLLL*. The corresponding Gray code *g*_*0*_ is 111,000,111,100, and *g*_− 1_ and *g*_+ 1_ are 111,000,111,101 and 111,000,110,100, respectively. The estimated value of *X*_*0*_ is (0.898187, 0.898423). But we took *X*_0_ as 0.232323. Therefore, this attack is unsuccessful in the case of the Superior Baptista Algorithm, as *X*_*0*_ does not fall within the calculated range.

Also, in the case of an alternate Superior Baptista Algorithm, we cannot perform this attack because.

(i) Corresponding to *r_even* = 4.8034, the c value is -3.366, and to *r_odd* = 4.76, the *c* value is -3.2844.
(ii) The range of *c* for the alternate Superior algorithm is [− 2.85, 2.85], so the values in (i) are out of range.
(iii) Since there are 2 *c* values, we can get two separate ranges for the ambiguous *X*_*0*_ value.

Through a specific procedure, we can determine that the initial key, i.e., *X*_0_, can be easily estimated using the initial value attack on the ciphertext generated by the Baptista algorithm, provided that only some initial ciphertext is available. However, the attack is ineffective on ciphers obtained by the Superior and Alternate Superior Algorithms.

#### Parameter estimation

In this cryptanalysis, we assume that we know the value of *X*_0_ and are interested in knowing the value of *c*. The value of *c* is estimated using the method described in section “Initial value estimation” and through additional calculation steps. For example, to construct the sequence of a dynamical system for orbit = *RL* (Gray code would be 01) and estimate the value of *c* as follows^14^: + √(-*c*-√-*c*) = *x*_0_, -*c* = √(-*c*) + *x*_0_^2^ by using the general formula mentioned in (11).

11 $$--{c_{{\text{n}}+{\text{1}}}}=\surd \left( { - {c_n}} \right)\,+\,{x_0}^{2}$$

We can approximate the original *c* value by putting *c*_0_ = 0 and then iterating the (8). After a certain point, we get a saturation point where *c*_*n*+1_ = *c*_n_. Using (8), we get a *c* value of -1.801779 for the Baptista algorithm. Thus, the key is broken. For the Superior algorithm at *β* = 0.9, we obtain a c value of -1.4474, whereas the key *c* value was − 2.1525. The alternative Superior algorithm has two *c* values based on *r_even* and *r_od*d values, but only one *X*_0_. This attack does not apply to this algorithm.

#### Comparative analysis with state-of-the-art schemes

We compared our proposed algorithms with several cutting-edge chaotic encryption systems to confirm their efficacy. Essential features, including chaotic dynamics, entropy levels, iteration counts, and resistance to different cryptanalytic assaults, are compiled in Table 10. Evidently, the original Baptista algorithm, based on only one logistic map, has low entropy (0.49) that can be attacked using key recovery, high-entropy attacks, etc. Likewise, both the hybrid logistic-tent scheme and the 2D chaotic map-based scheme have a medium entropy value (~ 0.60–0.70) and have not been significantly compromised by any attacking technique, such as differential analysis. Conversely, the suggested Superior Baptista Algorithm combines the Superior logistic map with an 8-parameter model, has a greater entropy (0.70), broader key space, allows greater resistance towards entropy depreciation and key recovery attempts, and has a maximum iteration number of 2,823. The final security enhancement performed through the Alternated Superior Baptista Algorithm is the combination of two chaotic attractors and the inherent use of Parrondo’s Paradox, which allows for the highest entropy (0.82) and represents a significantly greater computational complexity, as demonstrated by the number of iterations, which is 86,556. It exhibits the highest level of resistance to all assessed vectors of attack. In summary, both of these proposed schemes present promising values in terms of encryption soundness and unpredictability compared to current practices.

**Table 10:** Comparison of proposed algorithms with state-of-the-art schemes.

Scheme	Chaotic dynamics used	Key space expansion	Entropy value	Max iterations	Resistance to attacks
Baptista Algorithm^23^	Single Logistic Map	Low	0.49	~ 890	Vulnerable to key recovery, entropy, and one-time pad attacks
Hybrid Logistic-Tent^16^	Combined chaotic maps	Moderate	~ 0.60	N/A	Weak against differential analysis
2D Chaotic Maps^19^	2D Logistic or Arnold Cat Maps	Moderate	~ 0.70	N/A	Strong for images, weak for streams
Superior Baptista (Proposed)	Superior Logistic Map + β	High	0.70	2,823	Resistant to entropy & key recovery
Alternated Superior Baptista (Proposed)	Two Chaotic Attractors + Parrondo’s Paradox	Very High	0.82	86,556	Most substantial resistance to all tested attacks

## Limitations

The Superior Baptista and Alternated Superior Baptista Algorithms proposed in this work introduce improved chaotic properties, enlarged key spaces, and enhanced encryption complexity. However, these benefits come with certain limitations. Most notably, the increased number of iterations, particularly in the alternating version, results in higher computational overhead, thereby reducing efficiency in real-time or resource-constrained environments. Furthermore, the reliance on precise floating-point arithmetic can introduce implementation challenges on hardware platforms with limited precision or inconsistent numerical standards. The complexity of key structures involving multiple parameters (e.g., *β*, *r*_1_, *r*_2_) also increases the difficulty of key management and secure distribution. Synchronization between communicating parties can become sensitive to error propagation, especially in the presence of channel noise or transmission delays. Lastly, while the proposed methods strengthen resistance against classical attacks, comprehensive cryptanalysis under advanced attack models (such as differential, linear, or statistical cryptanalysis) remains an open area for further validation.

## Conclusion

The paper examines the security issues of Baptista’s chaotic cryptosystem, which various researchers have raised. In this paper, we focus on two problems with the Baptista algorithm: the non-uniform distribution of chaotic data and the reliance on a single attractor. We integrate Parrondo’s paradox. The Superior orbit and the logistic map in the Baptista algorithm introduce additional parameters to the secret key, thereby increasing the key space in the cryptosystem. The two proposed methods utilize more uniform chaotic data and two chaotic attractors, respectively, thereby increasing the security of the Baptista cryptosystem by employing a high number of iterations for encryption. Experimental evaluation revealed that the Superior Baptista Algorithm required up to 2,823 iterations, while the Alternated Superior Baptista Algorithm reached as high as 86,556 iterations, indicating significantly enhanced encryption complexity and diffusion. Furthermore, it was observed that the original Baptista algorithm is vulnerable to key recovery attacks, including parameter estimation and initial value estimation, entropy attacks, and one-time pad attacks. Entropy analysis showed marked improvement, with the Alternated Superior variant achieving an entropy value of 0.82, compared to 0.49 in the original Baptista algorithm, confirming greater randomness and unpredictability. Security testing against key recovery, entropy, and one-time pad attacks demonstrated that both enhanced algorithms were resistant to parameter and initial value estimation, and the effectiveness of entropy attacks was substantially reduced. We applied all these attacks to the two proposed algorithms and found that they are not vulnerable to any of these attacks. Specifically, the algorithms are not susceptible to key recovery attacks, the entropy attack is not very successful, and they are vulnerable to a one-time pad attack. While the one-time pad attack remains a theoretical concern due to interval-based symbol mapping, the use of dual chaotic attractors in the alternated version makes such attacks significantly less practical. Finally, we conclude that the Superior Baptista Algorithm and the alternative Superior Baptista Algorithm perform substantially better, overcoming most of the limitations of the original Baptista algorithm, including enhanced security against attacks. The alternate Superior Baptista Algorithm performs the best. It produces considerably more secure encryption than the Baptista algorithm and is robust against various attacks.

The work in the paper naturally raises the following curiosity: In Parrondo’s paradox, we chose two chaotic logistic maps. It would be interesting to see the Baptista cryptosystem’s results in three or more chaotic maps (using the Parameter Switching algorithm), which will be addressed in the future.

## Supplementary Information

Below is the link to the electronic supplementary material.

Supplementary Material 1

## Data Availability

All data generated or analysed during this study are included in this published article.

## List of symbols

*X*_0_: Initial value of the chaotic map; part of the secret key
*X*_n_: Chaotic state value at the *n*th iteration
*r*,* r*_1_,* r*_2_: Control parameters for the logistic map
*β*: Superior orbit parameter (0 < *β* ≤ 1)
*S*: Number of partitions for the attractor interval
*S*_i_: Individual partition/site in S representing a specific ASCII character
*Ε*: Length of each partition/site
*X*_min_,* X*_max_: Minimum and maximum bounds of the chaotic interval considered, respectively
*T*,* T*_i_: Plaintext message and its i-th character in the ASCII value, respectively
*C*,* C*_i_: Ciphertext message and its i-th element, respectively
*n*: Iteration counter
*O*(*f*,* X*_0_): Picard orbit of function *f* starting at *X*_0_
*SO*(*f*,* X*_0_, *β*_n_): Superior orbit for map *f*, starting at *X*_0_, with weighting sequence *β*_n_
*L*_1_,* L*_2_: Two logistic maps used alternately in the alternated Superior logistic map algorithm
*η*: Acceptance threshold used in probabilistic selection of chaotic values when multiple options exist.
*N*_0_,* N*_max_: Minimum and maximum allowed values for ciphertext *C*_i_, respectively
*k*: Random number between 0 and 1 used in encryption decision when *η* ≠ 0
*L*: Length of the plaintext message
*N_avg*: Average number of iterations required to find the desired partition
*H*(*S*): Entropy of symbol set *S*
*P*(*s*_i_): Probability of occurrence of symbol *s*_i_
*s*_1_,* s*_2_, …: Symbols used in entropy/key recovery analysis
*c*: Parameter in Mandelbrot/quadratic map used for key recovery attack analysis
*O*: Orbit of the logistic map
*g*_0_,* g*_+1_,*g*_-1_: Gray code sequences used in key recovery attack
*w*: Cipher array in key recovery attack
*f(x)*: Chaotic function
