# Supplementary material for: Mitigating security flaws in Baptista’s chaotic cryptosystem through superior and alternated logistic map approaches
Source: Sci Rep. 2025 Nov 3;15:38342. doi: 10.1038/s41598-025-22269-8 (PMC12583669; doi:10.1038/s41598-025-22269-8)
Supplement: Supplementary file 1 — Supplementary Material 1 [file 41598_2025_22269_MOESM1_ESM.docx]

**APPENDIX**

The program in “C” for the alternated superior Baptista cryptosystem follows.

#include<stdio.h>

#include<stdlib.h>

void main()

{

char plaintext[5]="hello";

int ascitext[5]={104,101,108,108,111}, ciphertext[5];

int m = 5, i, s=256;

long dec_asc[5], j, n;

float r_odd = 4.76, r_even=4.8034, xmin=0.32, xmax=0.98, e, eta=0, beta=0.7, k;

double x0=0.23232300000000,x00,r2,r1,xn;

x00 = x0;

e=(xmax-xmin)/s;

for(i=0;i<m;i++)

{

r2 = xmin+ascitext[i]*e;

r1 = xmin+(ascitext[i]-1)*e;

n=0;

while ( (n<250)||(n<=65532) )

{

if (n%2== 0)

xn=r_odd*x0*(1-x0)*beta+x0*(1-beta);

else

xn=r_even*x0*(1-x0)*beta+x0*(1-beta);

x0=xn;

n++;

if ( (n>=250) && (xn>=r1) && (xn<r2) )

{

if (eta==0)

{

ciphertext[i]=n;

break;

}

else

{

k = 0.1*random(10);

if (k>=eta)

{

ciphertext[i]=n;

break;

}

}

}

}

}

for(i=0;i<m;i++)

print("\n%d\n",ciphertext[i]);

// Decryption

print ("Decryption in ASCII\n");

for(i=0;i<m;i++)

{

for(j=0;j<ciphertext[i];j++)

{

if (j%2== 0)

xn=r_odd*x00*(1-x00)*beta+x00*(1-beta);

else

xn=r_even*x00*(1-x00)*beta+x00*(1-beta);

x00=xn;

}

for (s=256;s>0;s--)

{

r2 = xmin + s*e;

r1= xmin + (s-1)*e;

if ( (xn >=r1) && (xn <=r2) )

{

dec_asc[i]=s;

break;

}

}

}

for(i=0;i<m;i++)

printf("%d\n",dec_asc[i]);

}
